# Feeding on an exotic host plant enhances plasma levels of phenoloxidase by modulating feeding efficiency in a specialist insect herbivore

**DOI:** 10.3389/fphys.2023.1127670

**Published:** 2023-02-24

**Authors:** Carmen Mo, Angela M. Smilanich

**Affiliations:** ^1^ Department of Biology, University of Nevada, Reno, NV, United States; ^2^ Ecology, Evolution, and Conservation Biology Graduate Program, University of Nevada, Reno, NV, United States

**Keywords:** lepidoptera, herbivore, immunocompetence, nutrition, immnune response

## Abstract

**Background:** Exotic plant species represent a novel resource for invertebrates and many herbivorous insects have incorporated exotic plants into their diet. Using a new host plant can have physiological repercussions for these herbivores that may be beneficial or detrimental. In this study, we compared how using an exotic *versus* native host plant affected the immune system response and feeding efficiency of a specialist lepidopteran, the common buckeye (*Junonia coenia*: Nymphalidae, Hübner 1822).

**Materials and Methods:** In a lab experiment, larvae were reared on either the exotic host plant, *Plantago lanceolata* (Plantaginaceae), or the native host plant, *Mimulus guttatus* (Phrymaceae). Beginning at second instar feeding efficiency data were collected every 2 days until fifth instar when immune assays were performed. Immune assays consisted of standing phenoloxidase activity, total phenoloxidase activity, and melanization.

**Results:** Interestingly, we found that all three immune system parameters were higher on the exotic host plant compared to the native host plant. The exotic host plant also supported higher pupal weights, faster development time, greater consumption, and more efficient approximate digestibility. In contrast, the native host plant supported higher efficiency of conversion of ingested and digested food. The relationship between immunity and feeding efficiency was more complex but showed a large positive effect of greater host plant consumption on all immune parameters, particularly for the exotic host plant. While not as strong, the efficiency of conversion of digested food tended to show a negative effect on the three immune parameters.

**Conclusion:** Overall, the exotic host plant proved to be beneficial for this specialist insect with regard to immunity and many of the feeding efficiency parameters and continued use of this host plant is predicted for populations already using it.

## 1 Introduction

The colonization of exotic host plants by native herbivores is becoming a common phenomenon in insect communities (Graves and Shapiro, 2003; Jahner et al., 2011; Morrison and Hay, 2011; Yoon and Read, 2016). Understanding the mechanisms that lead to the colonization of novel host plants by herbivorous insects remains elusive despite many outstanding hypotheses and discourse on this topic (Agosta, 2006; Janz and Nylin, 2008; Forister and Wilson, 2013; Hardy and Otto, 2014; Mason, 2016). The consequences of using introduced host plants have been recently summarized in a meta-analysis by Yoon and Read (2016). This quantitative synthesis of the literature showed that lepidopterans overwhelmingly suffered reduced performance on exotic host plants compared to native host plants. As highlighted in this meta-analysis, most performance measurements were taken as growth, development, survival, and oviposition behavior. An important measure of performance that is often overlooked in these types of studies is the immune response. Since the immune response is one of the most effective defenses against natural enemies (Smilanich et al., 2009b), variation in its strength could provide valuable information regarding the consequences of using introduced host plants.

Ecoimmunology is a growing field of study that takes an integrative approach to explore the immunological aspects behind the relationship between organismal biology and ecology (Zuk and Stoehr, 2002; Schulenburg et al., 2009). Studies that focus on investigating the interaction between immunological function and ecological processes are part of this field (Schulenburg et al., 2009; Martin et al., 2011). Evidence from ecoimmunology shows that the immune system is energetically demanding, which can have costs for life history traits such as reproductive fitness and development (Lochmiller and Deerenberg, 2000; Schmid-Hempel and Ebert, 2003; Schmid-Hempel, 2005). Defending against infection while maintaining normal metabolic function can be costly (Schmid-Hempel and Ebert, 2003); however, these costs may be ameliorated when feeding on certain host plants, potentially providing opportunity for colonization of a new host plant (Resnik and Smilanich, 2020).

To understand the modulation of the immune response by host plant diet, it is imperative to investigate how herbivores are consuming and digesting host plant vegetation since both immunity and digestion are physiological processes that incur energy costs (Scriber and Slansky, 1981) and have been shown to influence each other (Becker et al., 2010; Varma et al., 2014). For example, Adamo et al. (2010) demonstrated physiological trade-offs between immunity and digestion by showing that immune-challenged crickets exhibited anorexic-like behavior, eating less food and also preferring food with low lipid content. To understand energy allocation, a classic approach is the Waldbauer feeding efficiency indices, which have been used to describe how efficiently animals process ingested food and convert it to body mass (Waldbauer, 1968). These indices have provided insight into how well herbivores are able to digest and process plants with differing concentrations and profiles of primary and secondary metabolites (Fox and Macauley, 1977; Cornell and Hawkins, 2003), and to a lesser extent how they are related to the immune response (Smilanich et al., 2009a; Smilanich et al., 2011). Smilanich et al. (2009a) found that high values of approximate digestibility (i.e., the amount of ingested food that crosses the gut) had a negative effect on the immune response of the caterpillar. In this case, the authors suggested that plant secondary metabolites that were crossing the gut within the host plant leaf material may have been interfering with the immune response (Smilanich et al., 2009a). Given the limited research investigating the association between immunity and feeding efficiency (but see Ponton et al., 2013), we sought not only to characterize this relationship, but also compare it between two different host plants.

Here, we focused on the lepidopteran immune system as a response that could shed light on the question of novel host use in insect herbivores. Since lepidopteran larvae spend a significant amount of time feeding and processing host plant material, we also investigated whether ingestion and digestion of host plant vegetation could have an impact on immune strength. We used the specialist herbivore, *J. coenia* (Lepidoptera: Nymphalidae), and two of its host plants, the exotic host plant (*Plantago lanceolata*: Plantaginaceae) and the native host plant (*Mimulus guttatus*: Phrymaceae). This specialist nymphalid caterpillar has recently incorporated *P. lanceolata* as a host plant (Robinson, 2002), and prior studies investigating the immune response of *Junonia coenia* have found that immunocompetence is host plant dependent and influenced by the secondary metabolites, iridoid glycosides, which are found in *P. lanceolata* (Smilanich et al., 2009a; Smilanich et al., 2018), but not found in *M. guttatus*. A recent study by Carper et al. (2019) compared the immune response *J. coenia* across developmental instars when reared on *P. lanceolata* and *M. guttatus.* They found that immune strength differed by instar and by host plant. Specifically, while the immune response was higher on *P. lanceolata* at third and fourth instars, the pattern changed at fifth instar with immunity higher on *M. guttatus*. The objectives of this study were three-fold: 1) compare the immunocompetence of *J. coenia* when reared on a native and exotic host plant, 2) determine the relationship between feeding efficiency and immunity, and 3) determine whether the relationship between feeding efficiency and immunity is host plant dependent.

## 2 Materials and methods

### 2.1 Study system

*Caterpillars and host plants*—The common buckeye (*J. coenia*) is a specialist nymphalid species that inhabits the southern United States and Mexico with migration into northern states during the summer (Robinson, 2002). The larvae primarily feed on plants containing iridoid glycosides (IGs), which is a secondary plant metabolite that acts as a feeding-stimulant, oviposition cue, and sequestered to deter natural enemies (Bowers, 1984; Pereyra and Bowers, 1988; Camara, 1997; Theodoratus and Bowers, 1999). The plant families containing IGs utilized by the buckeye are Scrophulariaceae, Plantaginaceae, Verbenaceae, and Acanthaceae (Bowers, 1984).

At our study site in Yuba Gap, California, the native host of the buckeye is the yellow monkeyflower (*M. guttatus*: Phrymaceae). *Mimulus guttatus* is a perennial and facultative annual plant that contains phenylpropanoid glycosides (PPGs), one of which, verbascoside, was determined to be a feeding stimulant for buckeyes (Holeski et al., 2013). Unlike with IGs, Holeski et al. (2013) showed that verbascoside was not sequestered by the larvae feeding on an artificial diet. However, it is unknown whether derivatives of verbascoside are sequestered. The levels of verbascoside in the leaves vary depending on the plant’s life history strategy with annual plants containing higher vebascoside concentrations (Holeski et al., 2013).

The non-native host plant at this study site is the narrowleaf plantain (*P. lanceolata*: Plantaginaceae). The buckeye has incorporated *P. lanceolata* into its host diet breadth after its colonization of North Americas 200 years ago (Thomas et al., 1987). *Plantago lanceolata* contains two iridoid glycosides, aucubin and catalpol in relatively equal amounts at concentrations that range between 5%–12% dry weight total IGs (Cavers et al., 1980; Bowers, 1984; Bowers and Collinge, 1992). *Plantago lanceolata* has been shown to be a high-quality host plant for the buckeye by positively influencing larval development and growth, which confer fitness consequences during adulthood (Bowers, 1984; Nieminen et al., 2003).

Both the native and novel host are available to the local buckeye population. We collected adults from this site and reared their offspring in the lab on either the assigned native or novel host to measure performance on each host. The common buckeye (*J. coenia*) is a specialist nymphalid species that inhabits the southern United States and Mexico with migration into northern states during the summer (Robinson, 2002). The larvae primarily feed on plants containing iridoid glycosides (IGs), which is a secondary plant metabolite that acts as a feeding-stimulant, oviposition cue, and sequestered to deter natural enemies (Bowers, 1984; Pereyra and Bowers, 1988; Camara, 1997; Theodoratus and Bowers, 1999). The plant families containing IGs utilized by the buckeye are Scrophulariaceae, Plantaginaceae, Verbenaceae, and Acanthaceae (Bowers, 1984).

### 2.2 Experimental design

**FIGURE 1:**
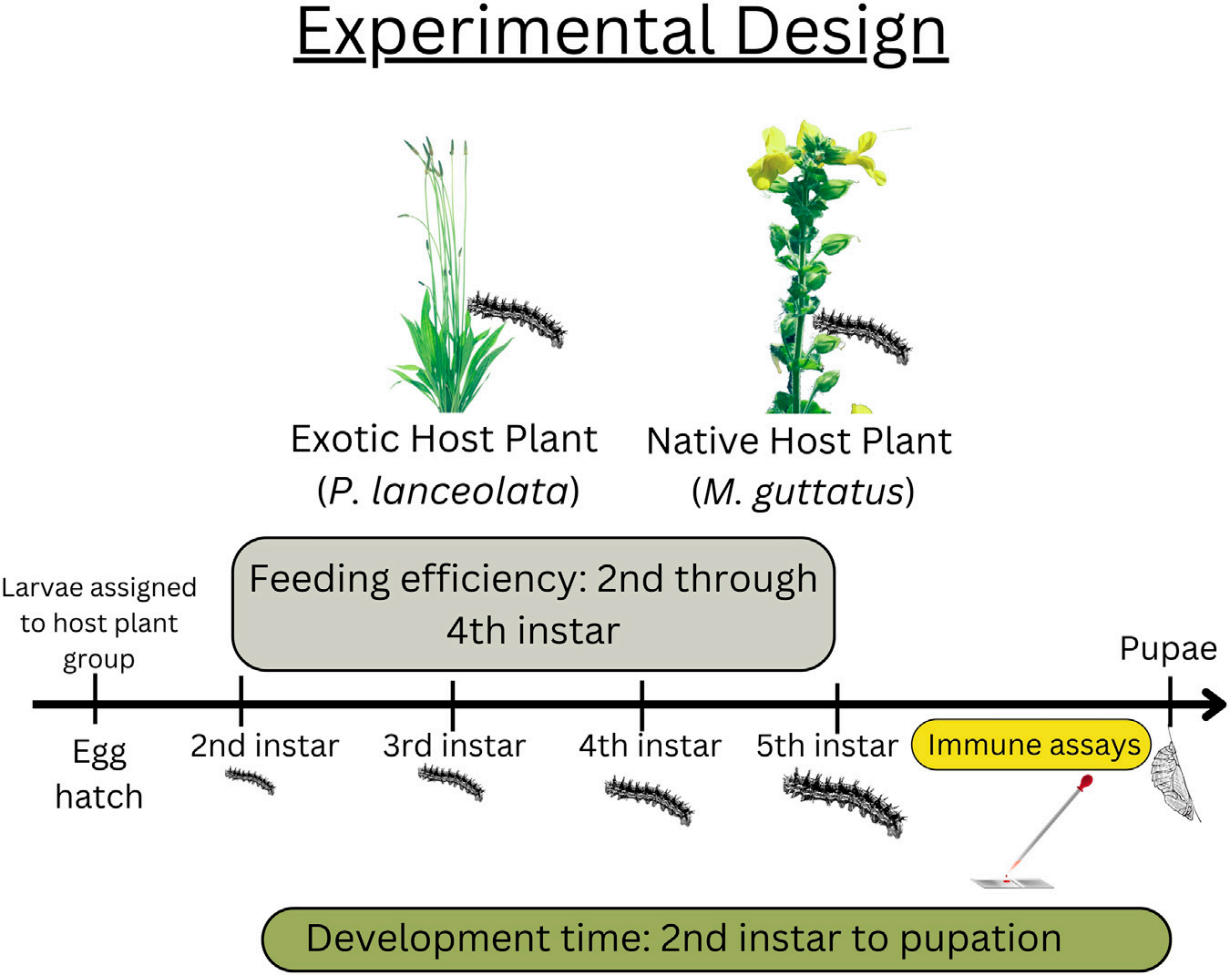
Experimental design including data collection timepoints. Feeding efficiency took place between the second and fourth instars. Data for the immune assays were collected at the beginning of the fifth instar. Development time was recorded from the beginning of second instar to pupation date.

Offspring from adults captured during summer 2015 were used in this study (see Figure 1 for experimental design). All larvae and adults were housed in a growth chamber (Percival) at 16 h light, 25 C:8 h dark, 20 C. Eggs were hatched on host plant material, then transferred to individual 2oz. Soufflé cups and assigned to either the native (*M. guttatus*) (*n* = 60) or novel host plant (*P. lanceolata*) (*n* = 60). Larvae were given fresh leaves every other day until second instar when the feeding efficiency trials began (see below). *Plantago lanceolata* plants were obtained from a local population in Idlewild Park, Reno, NV and *M. guttatus* plants were obtained from the study site in Yuba Pass, California. Pupal weights were measured once the pupal case had hardened (usually 24 h after pupation). Development time was analyzed as total development time (egg hatch to pupal date).

### 2.3 Feeding efficiency

The feeding performance of larvae on each host plant was investigated by collecting the weights of the larvae, frass, and the plant foliage every second day beginning at second instar and ending after molting into fifth instar. All wet weights were converted to dry weights prior to analyses using wet to dry weight conversion factors. The following gravimetric indices were then calculated: consumption index (CI), approximate digestibility (AD), efficiency of conversion of ingested material (ECI), and efficiency of conversion of digested material (ECD), as described by Waldbauer 1968. The formulas for the feeding efficiency indices and their definitions are shown below:

Consumption Index (CI) = dry weight of food consumed/mean dry weight of insect throughout the experiment.

Approximate digestibility (AD) = (dry weight of food consumed—dry weight of frass)/dry weight of food consumed.

Efficiency of conversion of ingested food (ECI) = larval dry weight gain/dry weight of food consumed.

Efficiency of conversion of digested food (ECD) = larval dry weight gain/(dry weight of food consumed—dry weight of frass)

### 2.4 Immune assays

*Phenoloxidase Assay*. The phenoloxidase (PO) assay is used to infer the strength of the immune response by measuring the degradation of the substrate by the enzyme PO over time in hemolymph (Gonzalez-Santoyo and Cordoba-Aguila, 2012). A subset of each host plant treatment group (*n* = 30) were chosen to have hemolymph collected for the PO assay. On the fourth day of fifth instar, 10.0 µl of hemolymph was collected using a micropipette and sterile insect pin to puncture the integument at the base of the third proleg. The hemolymph was immersed into 500 µl of phosphate-buffered saline (PBS) (Sigma-Aldrich) solution. Next, 100 µl of the PBS-hemolymph mixture was divided between two wells of a 96-well polystyrene microplate (Fisher-Scientific): 1) PO activated at the time the samples were taken (standing PO) and 2) all available PO including the stored, non-activated enzymes (total PO). To activate stored PO, 10.0 µl of 10% cetylpyridinium chloride (CPC) (Sigma-Aldrich) was added to each total PO well, followed by a 20-min incubation period. To correct for this additional volume, 10 µl of water was added to each of the standing PO well. Afterwards, 200 µl of 5 mM dopamine solution (Sigma-Aldrich) was placed in every well to act as the substrate for phenoloxidase. The plate was then immediately placed in a microplate reader (Bio-Rad iMark Microplate Absorbance Reader) for 45 min with readings made every 30 s at a wavelength of 490 nm. The linear phase of the reaction (determined to be between 0 and 20 min) was used for all analyses. Data were extracted from the spectrophotometer using Microplate Manager (MPM) software (Bio-Rad v.6.3).

*Sephadex Bead Assay*. Melanization were measured by injecting Sephadex beads into the hemocoel of the larvae (*n* = 15 for each host plant treatment). Following bead preparation methods in Smilanich et al. (2009a), DEAE Sephadex-A25 chromatography beads (40–120 µm diam) (Sigma-Aldrich) were dyed with a 0.1% solution Congo Red Dye (Sigma-Aldrich) and left to dry in the hood before use. A 30-gauge needle (Sigma-Aldrich) fastened onto a syringe was used to administer 10 beads immersed in PBS. After hemolymph collection for the PO assay, larvae were injected with beads in the same wound site created during hemolymph collection or within a 5 mm radius of the wound site. Larvae were returned to their individual 2.0 oz cups and given 24 h to mount an immune response before being freeze-killed. Beads were recovered from dissected larvae and photographed using a dissecting microscope connected to a digital camera (Carl Ziess Discovery V.8, AXIOCAM Software, Oberkochen, Baden-Wurttenburg, Germany). Beads were photographed at ×80 magnification, and their red value was scored in Adobe Photoshop (v6.0; Adobe System Inc., San Jose, California, United States). The red value of the bead ranges from 0–255 with 0 = pure gray and 255 = pure red. The mean red value was obtained for each bead within a caterpillar and these values averaged to provide a red value score for each individual caterpillar. The mean red value was transformed into a percentage of melanization [1—(red value/maximum red value)] for ease of interpretation. With the transformed red value, the higher the value the darker the bead, which means more melanization and a stronger immune response (Rantala and Roff, 2007; Smilanich et al., 2009b).

### 2.5 Statistical analyses

All statistical analyses were performed in SAS Statistical Analysis Software v.9.4 (Cary Institute N.C., United States). All data were analyzed using Bayesian regression models with the ‘genmod’ procedure (PROC GENMOD) with the ‘bayes’ option. Values for all data were scaled to z scores prior to running the models. The models had a burn-in size = 2000, MC sample size = 10,000, and a normal prior distribution (mean = 0, SD = 10^6^). For feeding efficiency data, a separate model was run for each feeding efficiency parameter and included host plant effects (*P. lanceolata* vs *Mimulus guttatus*) on CI, AD, ECI, and ECD. Similarly, a separate model was run for each immune parameter and included host plant effects on total PO, standing PO, and melanization. Models with host plant effect on development time and pupal mass were also run. Finally, the effect of feeding efficiency parameters (CI, AD, ECI, ECD) on the strength of total PO, standing PO, and melanization was analyzed using multiple regression models for each immune parameter separately. Separate models were run for each host plant.

To summarize the output of models, we used the posterior probability means β) for each comparison (e.g., total PO on *P. lanceolata* vs *Mimulus guttatus*) and the 95% highest posterior density interval (HPDI) (McElreath, 2020). For figures summarizing models with host plant species as the independent variable, the *x*-axis displays the effect size (difference in means between host plants) of the response variables displayed on the *y*-axis. In models comparing the effect of host plant (i.e., where host plant is the independent variable), *M. guttatus* was used as the reference host plant. Thus, a positive effect size indicates that individuals reared on *P. lanceolata* had larger values than those reared on *M. guttatus* for the given response variable. In the same vein, a negative effect size indicates that individuals reared on *M. guttatus* had larger values than those reared on *P. lanceolata* for a given response variable. Effect sizes that are close to zero indicate little to no difference between the means of the groups being compared. For simplicity, we refer to *P. lanceolata* as having a positive or negative effect on the measured response variable. In the case of feeding efficiency effects on immunity, a mean value close to zero indicates no effect of the feeding efficiency parameter on the immune response. Positive and negative mean values indicate that the relationship between the two variables are positive or negative.

The 95% HPDI shows the narrowest portion of the posterior probability distribution corresponding to 95% of the response variable in the distribution (McElreath, 2020). Bayesian posterior probabilities (PP) were calculated for pairwise comparisons of host plant and for the effect of feeding efficiency on immunity (e.g., Fordyce et al., 2011; Forister et al., 2013; Smilanich et al., 2016). Using this approach, if the effect size for a particular set of categories (e.g., total PO for individuals reared on *P. lanceolata*) is greater than the effect size for a comparable level of categories (e.g., total PO for individuals reared on M. guttatus) for more than 95% of the 10,000 MCMC iterations, then the two effect sizes are considered to be highly different, or highly different from zero in the case of feeding efficiency effects on immunity (Fordyce et al., 2011; Forister et al., 2013; Smilanich et al., 2016).

## 3 Results

### 3.1 Host plant effects on immune response

**FIGURE 2:**
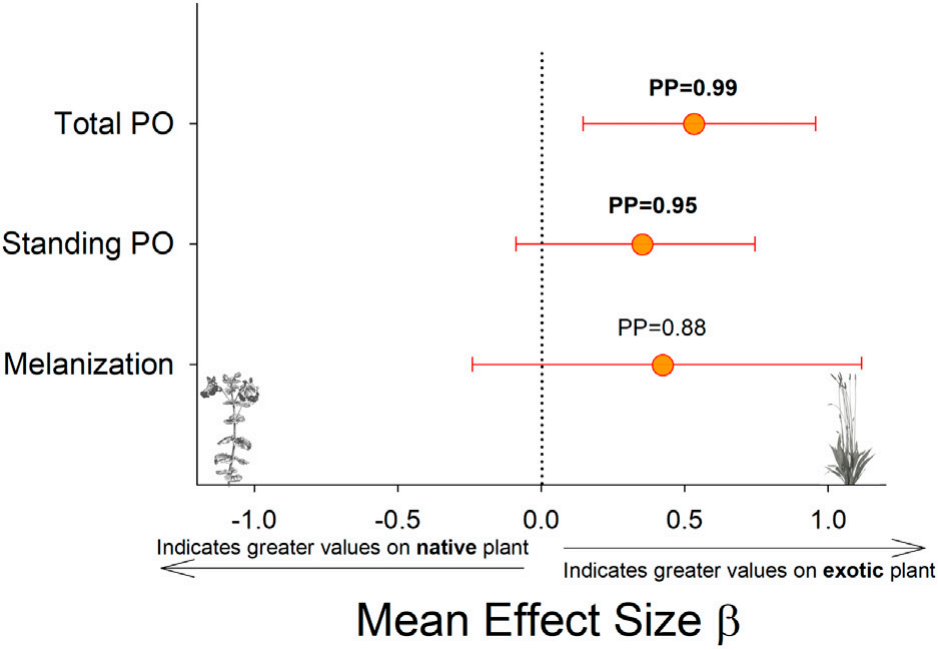
Comparison of each immune response measurement on the native host plant (*M. guttaus*) vs the exotic host plant (*Plantago lanceolata*). The mean effect size on the *x*-axis shows the difference in means between the two host plants for each immune response measurement. The native host plant is used as the reference condition in each comparison, thus positive mean effect size values indicate larger effects when reared on the exotic host plant, and negative mean effect size values indicate larger effects when reared on the native host plant. All response values have been transformed to z-scores. Bayesian posterior probabilities (PP) and 95% highest posterior density intervals (HPDI) are shown.

**FIGURE 3:**
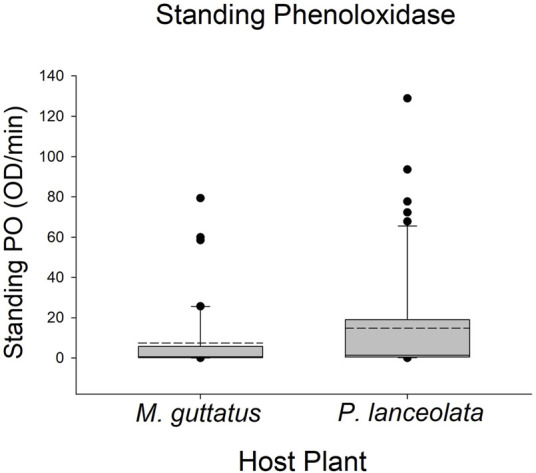
Boxplots representing the data for standing phenoloxidase activity for each host plant. Solid lines in the boxes represent the median standing PO and dotted lines represent the mean standing PO. Standing PO was higher on *Plantago lanceolata*.

**FIGURE 4:**
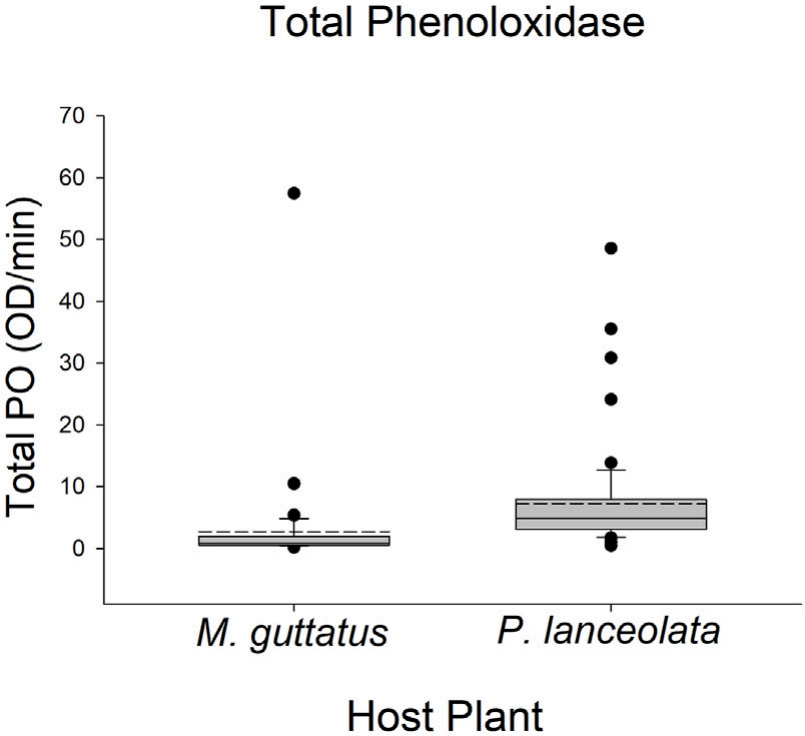
Boxplots representing the data for total phenoloxidase activity for each host plant. Solid lines in the boxes represent the median total PO and dotted lines represent the mean total PO. Total PO was higher in caterpillars reared on *Plantago lanceolata*.

**FIGURE 5:**
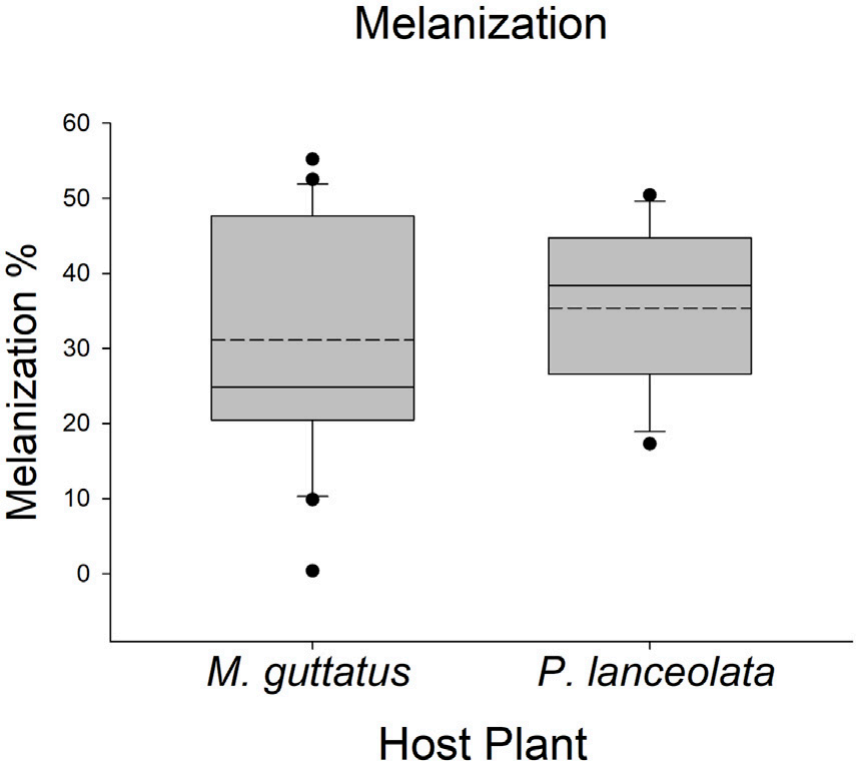
Boxplots representing the data for melanization for each host plant. Solid lines in the boxes represent the median melanization and dotted lines represent the mean melanization. Melanization was higher on *Plantago lanceolata*.

Larvae reared on *P. lanceolata* had higher standing PO (posterior probability mean β) = 0.36; highest posterior density interval (HPDI) = −0.05–0.78; posterior probability (PP) = 0.95, Figure 2; Figure 3) and total PO activity (β = 0.53; HPDI = 0.12–0.93; PP = 0.99, Figure 2; Figure 4) compared to individuals reared on *M. guttatus*. While the pattern was similar with melanization, the confidence was not as high (β = 0.42; HPDI = −0.28–1.1; PP = 0.88, Figure 2; Figure 5).

### 3.2 Host plant effects on feeding efficiency, development time, and pupal mass

**FIGURE 6:**
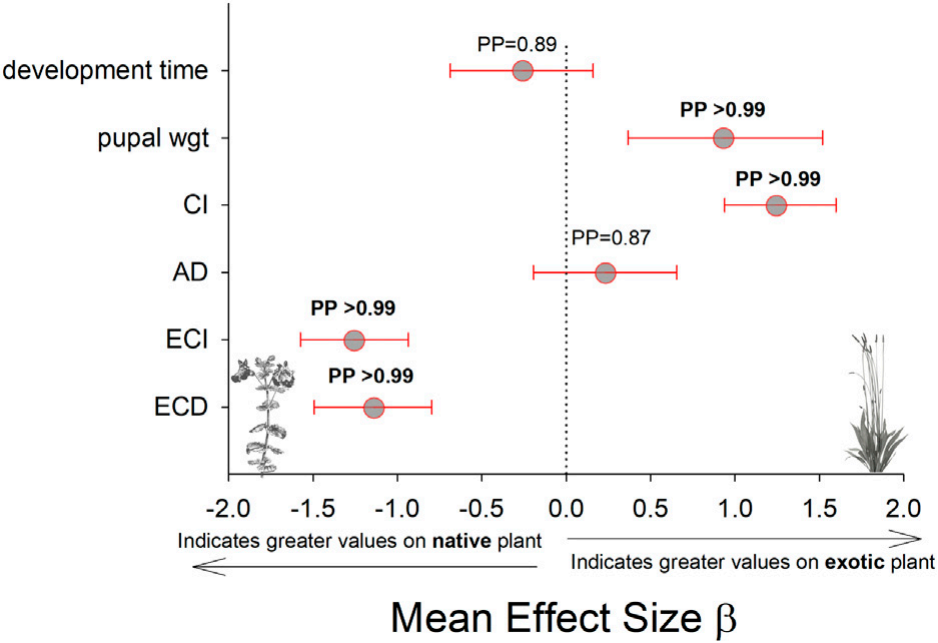
Comparison of development time, pupal mass, and feeding efficiency measurements on the native host plant (*M. guttaus*) vs the exotic host plant (*Plantago lanceolata*). The mean effect size on the *x*-axis shows the difference in means between the two host plants for each response variable. The native host plant is used as the reference condition in each comparison, thus positive mean effect size values indicate larger effects when reared on the exotic host plant, and negative mean effect size values indicate larger effects when reared on the native host plant. All response values have been transformed to z-scores. Bayesian posterior probabilities (PP) and 95% highest posterior density intervals (HPDI) are shown. CI = consumption index, AD = approximate digestibility, ECI = efficiency of conversion of ingested food, ECD = efficiency of conversion of digested food.

**FIGURE 7:**
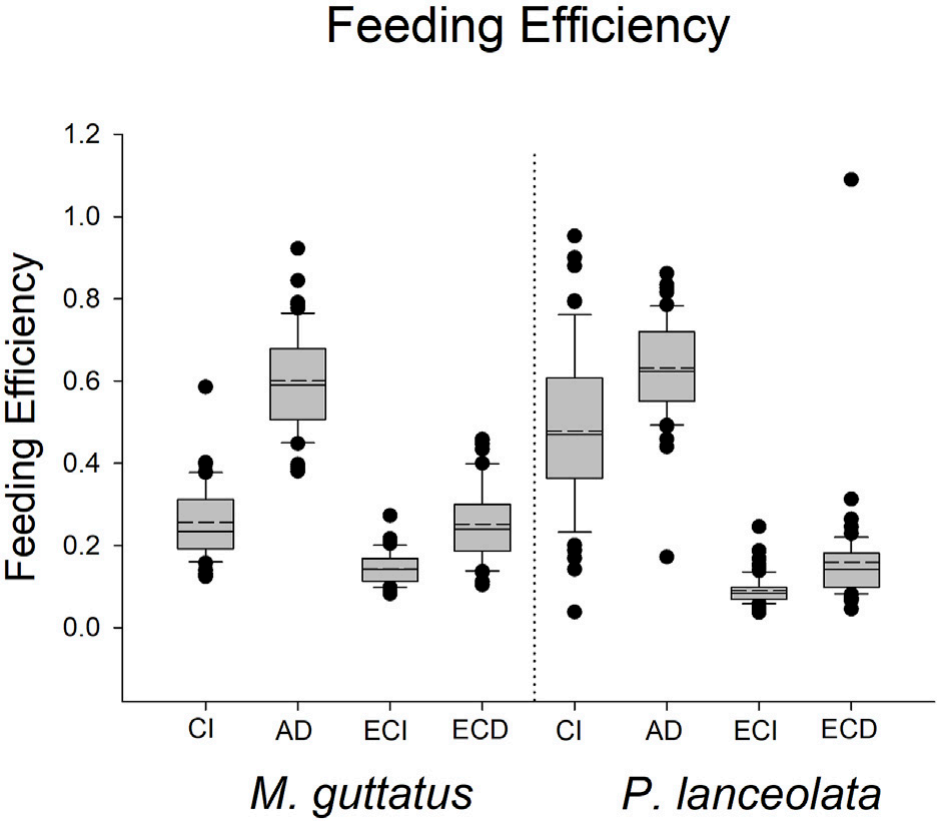
Boxplots representing the feeding efficiency data for each host plant. CI = consumption index, AD = approximate digestibility, ECI = efficiency of conversion of ingested food, ECD = efficiency of conversion of digested food.

The results for these three performance parameters were more mixed compared to the immune response results. Here we found that approximate digestibility (β = 0.23; HPDI = −0.20–0.64; PP = 0.87, Figure 6; Figure 7) and consumption index (β = 1.22; HPDI = 0.90–1.56; PP = 0.99, Figure 6; Figure 7) were greater when reared on *P. lanceolata*, while the efficiency of conversion of ingested food (β = −1.25; HPDI = −1.57—−0.91; PP = 0.99, Figure 6; Figure 7) and the efficiency of conversion of digested food (β = −1.14; HPDI = −1.49—−0.79; PP = 0.99, Figure 6; Figure 7) were reduced. In addition, development time was reduced when reared on *P. lanceolata* by 1 day (β = −0.26; HPDI = −0.67–0.14; PP = 0.89, Supplementary Figure S1). Pupal mass was an average of 60 mg greater for larvae reared on *P. lanceolata* (β = 0.92; HPDI = 0.32–1.45; PP = 0.99, Supplementary Figure S2).

### 3.3 Feeding efficiency and immunity

**FIGURE 8:**
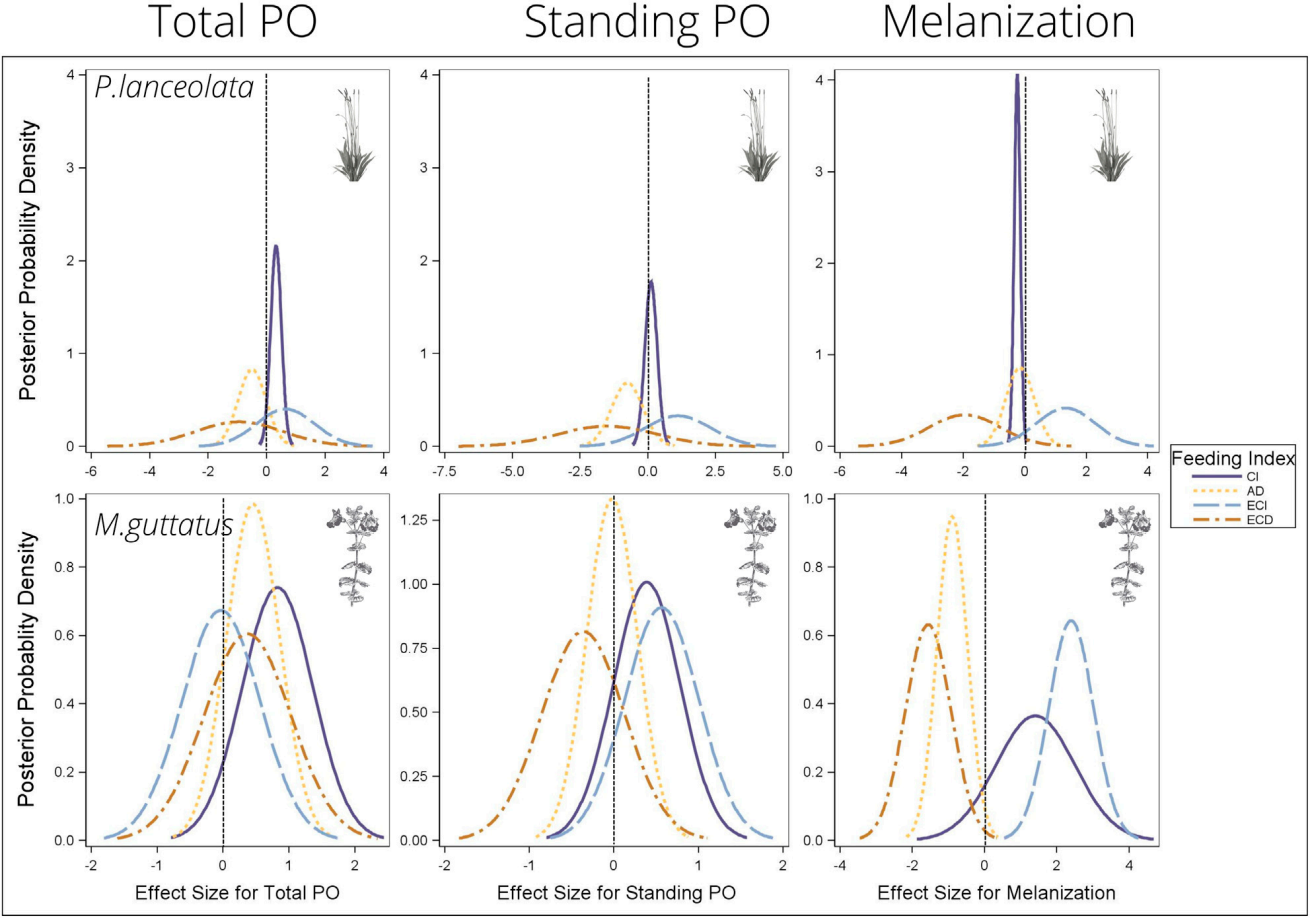
Bayesian posterior probability densities (PPDs) from the multiple regression analyses testing the effects of feeding efficiency measurements on the three immune response parameters. The *x*-axis shows the effect size for each feeding efficiency and immune response relationship. PPDs that are above zero indicate a positive relationship between feeding efficiency and immunity, and PPDs that are below zero indicate a negative relationship between feeding efficiency and immunity. See Table 1 for mean effect sizes, highest probability density intervals, and posterior probabilities. CI = consumption index, AD = approximate digestibility, ECI = efficiency of conversion of ingested food, ECD = efficiency of conversion of digested food.

*Consumption index*—For both host plants, higher consumption of vegetation was associated with higher standing and total PO, although the effect size was greater for individuals reared on *P. lanceolata* (see Figure 8 and Table 1 for all statistical summaries). Interestingly melanization was reduced with greater consumption of *P. lanceolata*, but not for individuals consuming *M. guttatus.*

**TABLE 1:** Statistical results of the regression analysis testing the effects of feeding efficiency measurements on the three immune response assays. The mean effect size and 95% highest posterior density interval (HPDI) for the three immune response parameters, total PO, standing PO, and melanization are shown. The Bayesian posterior probability (PP) represents the pairwise comparisons of each feeding efficiency measurement and immune response assay. Negative mean values indicate a negative relationship between the feeding efficiency parameter and the measured immune response, and likewise for positive mean values. Bolded numbers are PPs higher than 95%.

Host plant	Total PO		Standing PO		Melanization	
***P. lanceolata***	**Mean β (HPDI)**	**PP**	**Mean β (HPDI)**	**PP**	**Mean β (HPDI)**	**PP**
CI	**0.31 (−0.06–0.67)**	**0.95**	0.11 (−0.34–0.54)	0.70	**−0.24 (−0.44—-0.05)**	**0.99**
AD	−0.50 (−1.42–0.46)	0.86	**−0.77 (−1.89–0.32)**	**0.99**	−0.16 (−1.06–0.74)	0.65
ECI	0.62 (−1.35–2.55)	0.73	1.11 (−1.13–3.52)	0.82	1.33 (−0.56–3.11)	0.92
ECD	−0.94 (−3.88–2.01)	0.74	−1.52 (−5.02–2.03)	0.81	**−1.96 (−4.14–0.32)**	**0.99**
*M. guttatus*						
CI	0.81 (−0.22–1.86)	0.93	0.38 (−0.40–1.15)	0.83	1.40 (−0.80–3.47)	0.90
AD	0.44 (−0.38–1.21)	0.86	−0.02 (−0.63–0.56)	0.54	**−0.90 (−1.74—-0.06)**	**0.99**
ECI	−0.02 (−1.23–1.14)	0.52	0.56 (−0.30–1.42)	0.90	**2.39 (1.19–3.63)**	**0.99**
ECD	0.35 (−0.96–1.69)	0.70	−0.37 (−1.34–0.57)	0.81	**−1.56 (−2.87—-0.34)**	**0.99**

*Approximate digestibility*—For individuals feeding on *M. guttatus* higher approximate digestibility greatly reduced melanization but had no effect for individuals feeding on *P. lanceolata* (Figure 8; Table 1). For *M. guttatus* feeders, higher approximate digestibility increased total PO, while standing PO was not affected. For individuals reared on *P. lanceolata*, higher approximate digestibility reduced both total and standing PO.

*Efficiency of conversion of ingested food*—For individuals feeding *P. lanceolata*, greater ECI was weakly associated with a higher total and standing PO but had a stronger positive effect on melanization (Figure 8; Table 1). For individuals feeding on *M. guttatus,* greater ECI had no effect on total PO, a weak positive effect on standing PO and a strong positive effect on melanization.

*Efficiency of conversion of digested food*—For individuals feeding on *P. lanceolata*, greater ECD was weakly associated with lower total and standing PO but had a stronger negative effect on melanization (Figure 8; Table 1). For individuals feeding on *M. guttatus*, greater ECD had a weak positive effect on total PO and a weak negative effect on standing PO. However, for melanziation, there was a strong negative effect of high ECD.

## 4 Discussion

In this study, we sought to understand the immunological and digestive impacts of feeding on an exotic host plant compared to a native host plant for a specialist insect herbivore. Overall, we found higher immune performance in individuals reared on the exotic host plant (*P. lanceolata*) along with higher pupal weights, faster development time, and higher consumption index. While individuals reared on the native host plant (*M. guttatus*) had greater efficiency of conversion of ingested (ECI) and digested food (ECD), these two indices were only weakly associated with total and standing phenoloxidase activity and the ECD had a strong negative impact on melanization. Overall, our data find support for the hypothesis that *J. coenia* populations will continue using this exotic host plant and benefit in an immunological capacity compared with the native host plant.

### 4.1 Immune response and host plant

Individuals reared on *P. lanceolata* had stronger total PO and melanization compared to individuals reared on *M. guttatus*. These data support our prior findings that showed a stronger activation of the PO response when buckeyes were reared on *P. lanceolata* compared to another exotic host plant, *Plantago major* (Smilanich et al., 2018). In partial support of our results, Carper et al. (2019) found that the melanization response was higher on *P. lanceolata* compared to *M. guttatus*, but only at younger instars (third and fourth), while fifth instars had a higher melanization response on *M. guttatus*. Here, we measured the immune response during the fifth instar only. It would be enlightening to measure immunity and feeding across all the instars to determine whether the patterns that we found during the fifth instar are supported during their entire larval developmental stage. Since fifth instar is the final larval stage of the buckeye, there may be significant physiological changes occurring as the they prepare for pupation that could influence resource investment and thus the interaction between immunity and feeding efficiency (Awmack and Leather, 2002; Adamo et al., 2016).

*Plantago lanceolata* produces two iridoid glycosides, aucubin and catalpol, that are sequestered by buckeye larvae, while *M. guttatus* does not produce these same iridoid glycosides. Prior data show that high levels of sequestration of these two iridoid glycosides reduces the immune response (Smilanich et al., 2009a; Carper et al., 2019; Muchoney et al., 2022); non-etheless, our data here show that individuals reared on *P. lanceolata* outperform individuals reared on *M. guttatus*, indicating that *M. guttatus* may be especially detrimental for immunity. While *M. guttatus* does not contain aucubin or catalpol, it does contain phenylpropanoid glycosides, specifically, verbascoside, which has been shown to be a feeding stimulant for *J. coenia* larvae but is not sequestered (Holeski et al., 2013). In a broader sense, the impact of novel host plants on herbivore immunity may provide insight into how new host plants become incorporated into the diet breadth of herbivores (Smilanich et al., 2018; Muchoney et al., 2022).

### 4.2 Feeding efficiency

Larval development is heavily influenced by the nutritional value of the host (Awmack and Leather, 2002; Raubenheimer and Simpson, 2009; Ponton et al., 2013), thus in this framework, determining whether the native or exotic host plant yields better larval performance allow inferences to be made about nutritional quality. The nutritional indices, ECI and ECD, were higher on the native host plant, *M. guttatus*, compared to *P. lanceolata*, showing that individuals reared on their native host plant were more efficient at converting plant material into biomass. Even so, pupal mass was still higher on *P. lanceolata,* the exotic host plant. The greater consumption index (CI) values for larvae reared on the *P. lanceolata* may offer an explanation and suggests that high pupal mass can be attained simply by consuming more plant material, regardless of digestion efficiency. The greater CI values may also explain the trend that larvae developed faster on average when reared on the exotic host plant, despite there being no significant effect on development time. Lastly approximate digestibility (AD), which is the amount of consumed food that crosses the gut, was moderately higher for individuals reared on *P. lanceolata*, which is consistent with prior published data (Smilanich et al., 2009a). Considering feeding indices along with development time and pupal mass, *P. lanceolata* appears to provide superior nutritional support compared to the native host plant, *M. guttatus*.

### 4.3 Immune response and feeding efficiency

One dominant pattern that was found between the immune response and feeding efficiency was the positive association between the amount of plant material consumed and the immune response. This positive effect is most likely simply due to increased nutritional resources available for fueling normal metabolism including immunity. Several studies have found that increased access to nutritional resources is beneficial to the immune response, particularly access to protein (Povey et al., 2009; Ponton et al., 2013; Cotter et al., 2019; Wilson et al., 2019; Ponton et al., 2020). Although we did not investigate the nutritional resources within the host plants used in our study, we can assume that both offer some baseline access to nutrition for herbivores. However, the positive effect of consumption did not hold for the melanization response for individuals feeding on *P. lanceolata*. In this case, higher consumption was strongly associated with lower melanization. This could be due to these individuals allocating resources towards mass gain instead of melanization since we also found that a high efficiency of conversion of digested (ECD) food was negatively associated with melanization in this group. A high ECD indicates that individuals are efficiently turning digested food to caterpillar biomass, thus possibly prioritizing resources to gaining biomass over melanization. Moreover, since the efficiency of conversion of ingested food (ECI) was positively associated with melanization in this group, the putative resource diversion may be occurring after the plant material has been through the hindgut and postdigestion. Adamo et al. (2016) found that the immune response of *Manduca sexta* (Sphinididae) caterpillars undergoes a reconfiguration following nutritional stress in order to prioritize resources. In this information rich study, the authors found that when nutritional resources were scare, larvae prioritized constitutive immune defenses (PO cascade). In our study, we did not measure nutritional composition, but the possibility for immune reconfiguration based upon access to nutrition as shown by Adamo et al. (2016) highlights the real-time flexibility of the immune system in response to environmental conditions.

Beyond consumption, the patterns between immunity and feeding efficiency tended to be host plant dependent and strongest for melanization. This is most likely due to differences in how vegetation from the two host plants is processed post-consumption and during digestion. While approximate digestibility (AD) did not have a large effect on total or standing PO on either host plant, it had a strong negative association with melanization for individuals reared on the native host plant, *M. guttatus*, but no effect for individuals reared on *P. lanceolata*. Approximate digestibility measures how much plant material is crossing the midgut, and for individuals reared on *M. guttatus*, the more vegetation that crossed the gut, the lower the melanization response. As mentioned before, this negative association could be due to resources being diverted towards converting this greater amount of plant material to caterpillar biomass since ECD also showed a negative association in this group. Overall, it appears that there may be a resource trade-off between biomass gain and immunity, which has been shown in a number of insect species including bumblebees (Moret and Schmid-Hempel, 2000), fruit flies (Ayres and Schneider, 2009), and other species of Lepidoptera (Cotter et al., 2011; Povey et al., 2014).

It should be noted that our study did not encompass all immune parameters that compose the insect immune response. We focused on the phenoloxidase cascade, which has been shown to be an important immune response (Smilanich et al., 2009b; Gonzalez-Santoyo and Cordoba-Aguila, 2012), however, immune parameters do not always respond in the same way (Adamo, 2004; Wilson et al., 2019). Thus, it is possible that parameters such as hemocyte density, anti-microbial peptides, and lysozyme-like activity may be responding differently than the phenoloxidase cascade. In addition, a heightened phenoloxidase response has the potential to cause self-harm to the organism due to cytotoxic compounds that are produced during the production of melanin (Sadd and Siva-Jothy, 2006; Cornet et al., 2007). With regard to our results, regulation of the PO cascade may be different on the two host plants with larvae reared on *M. guttatus* displaying tighter regulation of the PO cascade to avoid self-harm and unwanted costs. Non-etheless, even with a heightened PO response, larvae reared on *P. lanceolata* still had higher pupal mass and faster development showing that at least for these two performance parameters there were no costs.

## 5 Conclusion

Overall, the results found here demonstrate that incorporation of an exotic host plant may be facilitated by the immune response of the herbivore, providing a clear mechanism for how novel host plant associations can begin and persist in natural populations. Future work should include additional metrics related to digestive health and performance such as the microbiome, which is at a frontier with regard to understanding how resident and passive microbes influence immunity (Wu et al., 2016; Smilanich et al., 2018; Yoon et al., 2019; Duplouy et al., 2020), especially within insect herbivores.

## Data Availability

The raw data supporting the conclusion of this article will be made available by the authors, without undue reservation.
