# Supplementary figures and images for: Feeding on an exotic host plant enhances plasma levels of phenoloxidase by modulating feeding efficiency in a specialist insect herbivore
Source: Front Physiol. 2023 Feb 24;14:1127670. doi: 10.3389/fphys.2023.1127670 (PMC9998540; doi:10.3389/fphys.2023.1127670)

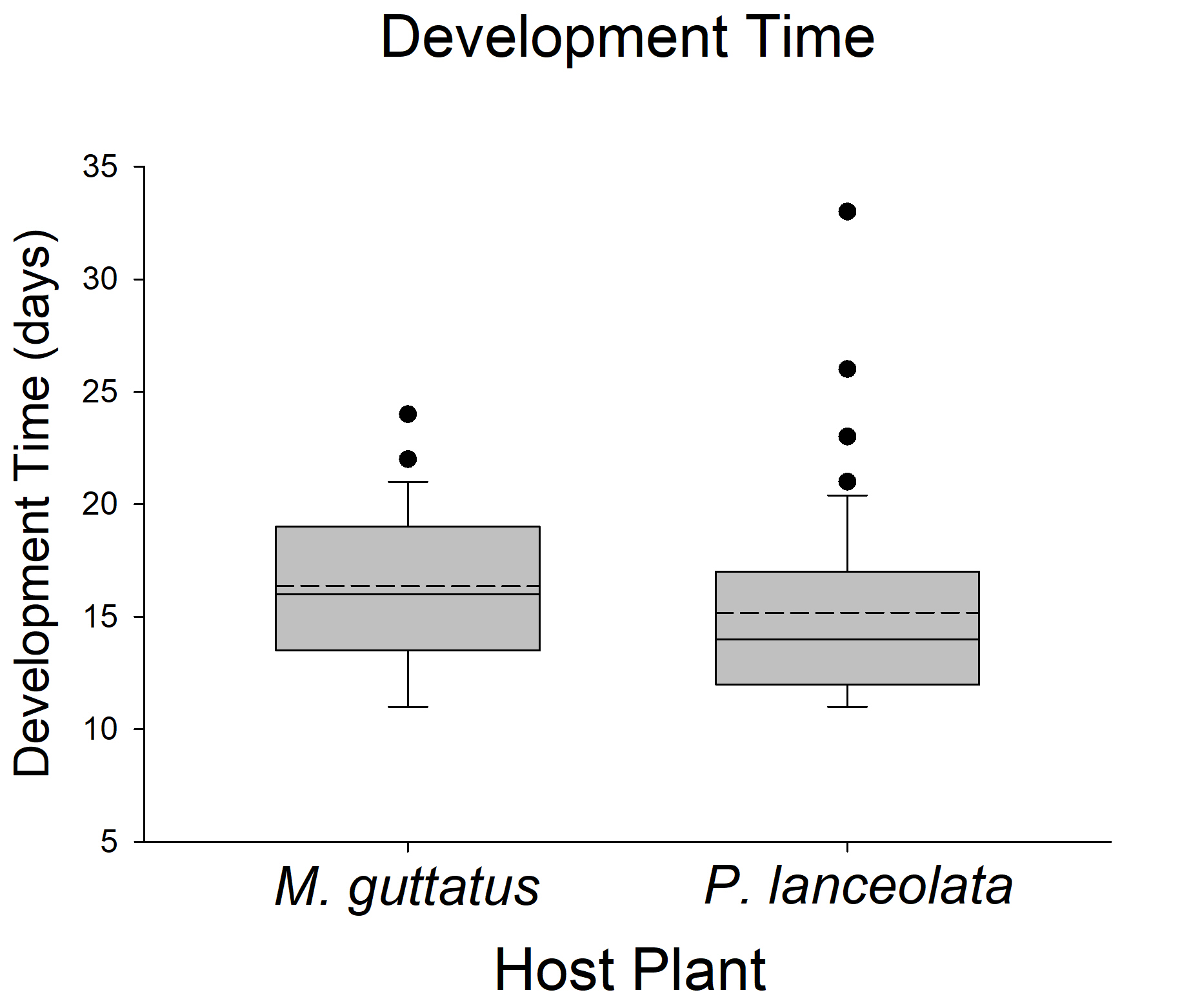

Supplement: Supplementary file 1 [file Image1.JPEG]

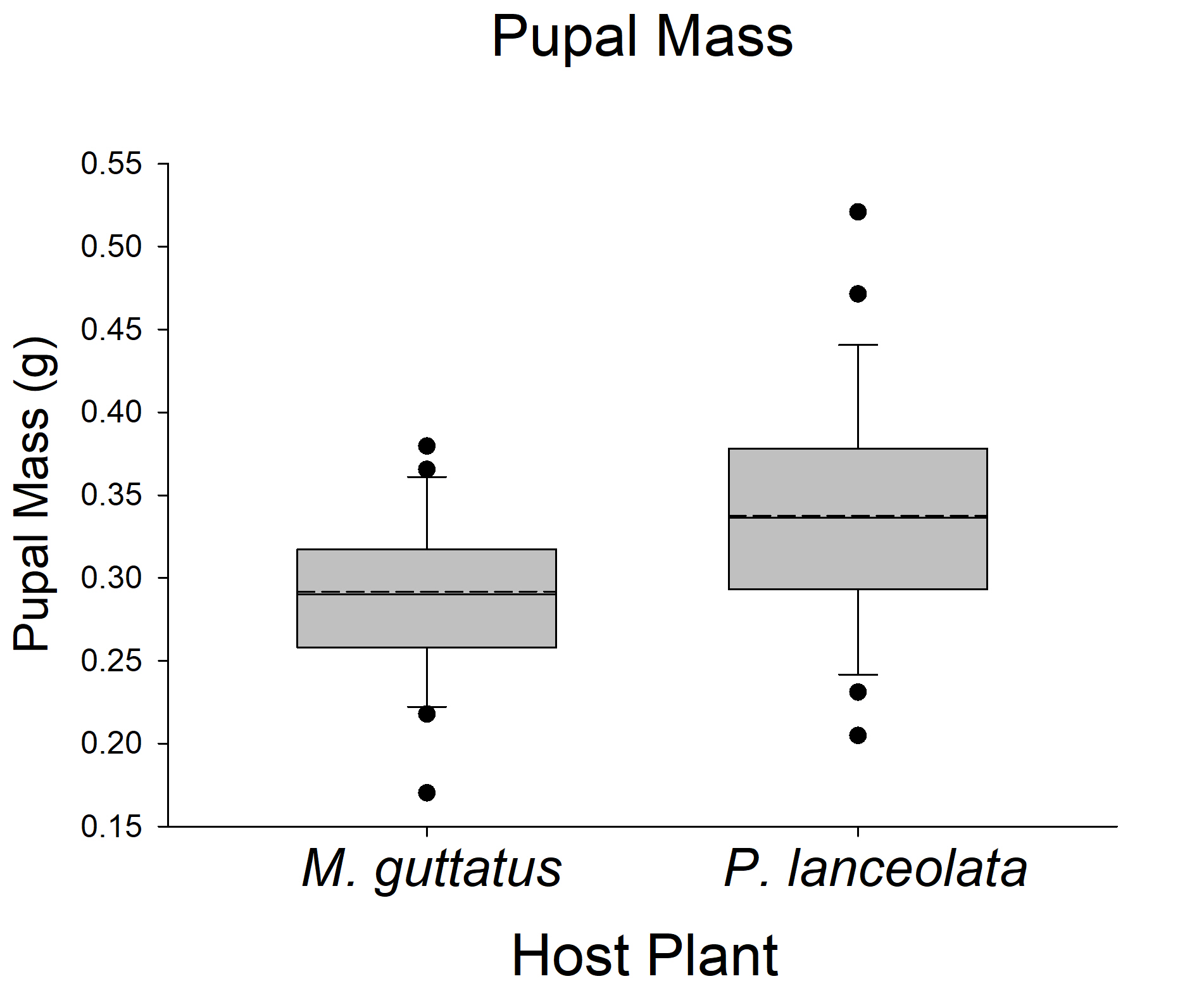

Supplement: Supplementary file 2 [file Image2.JPEG]
